# YTHDF2 is a Potential Biomarker and Associated with Immune Infiltration in Kidney Renal Clear Cell Carcinoma

**DOI:** 10.3389/fphar.2021.709548

**Published:** 2021-08-27

**Authors:** Ganglin Su, Tianshu Liu, Xiaohong Han, Hao Sun, Wenan Che, Kun Hu, Junwen Xiao, Yanfeng Li, Yuchen Liu, Wujiao Li, Hongbing Mei

**Affiliations:** ^1^ Department of Urology, the First Affiliated Hospital of Shenzhen University, Shenzhen Second People’s Hospital, Shenzhen, China; ^2^ Shantou University Medical College, Shantou, China; ^3^ Key Laboratory of Medical Reprogramming Technology, the First Affiliated Hospital of Shenzhen University, Shenzhen Second People’s Hospital, Shenzhen, China; ^4^ Guangdong Key Laboratory of Systems Biology and Synthetic Biology for Urogenital Tumors, the First Affiliated Hospital of Shenzhen University, Shenzhen Second People’s Hospital, Shenzhen, China; ^5^ Hunan Key Laboratory of Economic Crops Genetic Improvement and Integrated Utilization, School of Life Sciences, Hunan University of Science and Technology, Xiangtan, China

**Keywords:** YTHDF2, KIRC, biomarker, prognosis, immune infiltrates, N6-methyladenosine (m6A) RNA methylation

## Abstract

Clear cell renal cell carcinoma (ccRCC or KIRC) has a high mortality rate globally. It is necessary to identify biomarkers and investigate the mechanisms those biomarkers are associated with, to improve the prognosis of patients with KIRC. N6-Methyladenosine (m6A) affects the fate of modified RNA molecules and is involved in tumor progression. Different webservers were used in our research to investigate the mRNA transcription and clinical significance of YTHDF2 in KIRC. Survival analysis revealed that patients with elevated YTHDF2 transcription had a slightly longer OS and DFS than those with low YTHDF2 expression. YTHDF2 expression was shown to be significantly associated with the abundance of immune cells such as B cells, CD8^+^ T cells, CD4^+^ T cells, macrophages, neutrophils, and dendritic cells. For a series of enrichment studies, we combined information on YTHDF2-binding molecules and expression-linked genes and identified the possible influence of “mRNA surveillance pathway,” “RNA degradation,” and “RNA transport” in the biology or pathogeny of KIRC. In addition, we identified multiple miRNA, kinase, and transcription factor targets of YTHDF2 in KIRC and constructed target networks. Overall, our findings show that YTHDF2 is a possible indicator of immune infiltration in the KIRC.

## Introduction

KIRC is the third most common urinary system tumor, but it has the highest mortality rate (McDougal et al., 2015). It affects renal parenchymal cells and displays complex behaviors (Linehan et al., 2019). Currently, surgery is the main treatment strategy for KIRC, but is associated with a high risk of recurrence and metastasis (Chin et al., 2006). Radio and chemotherapies have similarly proved underwhelming. Immune therapy has revolutionized the treatment of cancer, and tumor immunology vigor (Riley et al., 2019). Immunotherapy has shown promising clinical effects in the treatment of renal cancer and has been a hot subject in the field (Xu et al., 2020). However, immunotherapy has a low rate of reaction (Rini and Atkins, 2009). To increase the response rate, reliable tumor immunotherapy biomarkers as targets or diagnosis and evaluation indicators will be beneficial. Investigations into identifying biomarkers of KIRC and developing therapeutic agents which influence their behavior, and ultimately results in earlier detection of KIRC, better treatments and patient prognoses are of great value for clinicians, oncologists, and of course KIRC patients.

N6-Methyladenosine (m6A) is a regular mRNA modification that was first identified and partially described in the 1970s (Adams and Cory, 1975; Beemon and Keith, 1977). Numerous recent studies have shown that m6A modification plays a key role in various types of carcinogenesis (Panneerdoss et al., 2018; Lan et al., 2019). Several studies have suggested a correlation between m6A and various cancers, including urologic neoplasms, digestive system neoplasms, and hematological cancer (Chen et al., 2018; Su et al., 2018; Chen et al., 2019; Han et al., 2019; Paris et al., 2019; Wang Q. et al., 2020).

“Writers,” “erasers,” and “readers” of the m6A gene are proteins that can add, delete, or identify m6A-modified sites and modify essential biological processes correspondingly (Wang et al., 2015; Śledź and Jinek, 2016; Meyer and Jaffrey, 2017).

Among the m6A reader proteins, YTHDF2 was the first to be recognized and studied extensively, affecting mRNA stability (Chandrashekar et al., 2017). YTHDF2 has been shown to affect tumor development in certain cancers by degrading m6A-modified mRNAs by binding to m6A sites. However, the role of YTHDF2 remains controversial, indicating that YTHDF2 is involved in a complex network of regulation in tumors. According to some research, YTHDF2 plays a significant function in pancreatic cancer (Chen et al., 2017). YTHDF2 binds RNA epitranscriptomic modifications to the development of glioblastoma stem cells (Dixit et al., 2021). YTHDF2 supports the cancer stem cell phenotype and metastasis in patients with liver cancer (Zhang et al., 2020). YTHDF2 was found to be upregulated in bladder cancer; upregulated YTHDF2 aids bladder cancer progression (Xie et al., 2020). However, some research indicates that YTHDF2 suppresses tumor progression in Hepatocellular carcinomas and osteosarcomas (Hou et al., 2019; Zhong et al., 2019; Yang et al., 2020).

The expression of YTHDF2 in primary KIRC and its association with relevant clinicopathological features and prognostic significance was investigated in this study.

For the first time, we created a predictive nomogram by combining relevant clinical factors and YTHDF2 gene expression. The immune penetration, genetic modification, functional states, relevant cellular pathway, and potential biological roles of YTHDF2 in KIRC progression were investigated using various web tools, which will aid in the understanding of a possible mechanism for kidney carcinogenesis. Additionally, we discovered YTHDF2 targets in the form of miRNA, kinase, and transcription factors in KIRC and constructed target networks. It is hoped that this study would lead to a better understanding of the possible role of YTHDF2 in tumor immunology and its prognostic value in KIRC.

## Methods

### Analyses of YTHDF2 Gene Expression

We used the “Gene DE” module of the TIMER2 (Tumor immune estimation resource, version 2) web (http://timer.cistrome.org/) to aim at the expression variation of YTHDF2 between tumor and neighboring normal tissues for the various tumors and tumor subtypes in the TCGA project. The TCGA database (https://tcga-data.nci.nih.gov/tcga/) was used to collect the gene expression profiles of primary KIRC patients (April 12, 2021). Data on sex, age, survival, and results were obtained from the TCGA database. UALCAN is a robust website for analyzing specific genes in a variety of ways (Chandrashekar et al., 2017). The “Expression Analysis” component of UALCAN was applied to determine the mRNA level of YTHDF2 in various subgroups of patients with KIRC. The Human Protein Atlas (HPA) database was used to analyze YTHDF2 protein expression in clinical specimens (Asplund et al., 2012).

### Analysis of the Prognosis for Survival

Disease-free survival (DFS) and overall survival (OS) significance map data of YTHDF2 through the TCGA KIRC dataset using the “Survival analysis” feature of GEPIA2 (Tang et al., 2019). Cutoff high (50%) and cutoff low (50%) values were used as expression levels in GEPIA2 to separate the high-expression and low-expression cohorts.

### Analysis of Genetic Alteration

The cBioPortal website (https://www.cbioportal.org/) was used for genetic alteration analysis (Cerami et al., 2012; Gao et al., 2013). In the “Quick select” segment, we selected “Kidney Renal Clear Cell Carcinoma (TCGA, Firehose Legacy)” and entered “YTHDF2” for queries on the genetic modification characteristics of YTHDF2. In mRNA expression z-scores (RNA Seq V2 RSEM) and protein expression z-scores, a threshold of 2.0, was set (RPPA). The “Cancer Types Summary” module displayed the effects of the modification frequency, mutation form, and CNA (Copy number alteration).

### Analysis of Immune Infiltration

TIMER is a convenient method for analyzing immune infiltrates in TCGA tumors (Li et al., 2017). Using the Wilcoxon test, the “Diff Exp” module was used to investigate YTHDF2 gene expression differences between tumor and normal tissues. The “Gene module” was used in our research to assess the relationship between YTHDF2 and immune cell infiltration.

### Analysis of YTHDF2-Related Gene Enrichment

Centered on the TCGA KIRC dataset, we used GEPIA2’s “Similar Gene Detection” module to find the top 100 YTHDF2-correlated targeting genes. We used a single protein name (“YTHDF2”) and organism (“*Homo sapiens*”) to scan the STRING database (https://string-db.org/) and acquired the YTHDF2-binding protein (Szklarczyk et al., 2019). The following key parameters were set: the minimum required interaction score [“Low confidence (0.150),” the meaning of network edges (“evidence”), the maximum number of interactors to display (“no more than 50 interactors” in the first shell), and active interaction sources (“experiments”)]. We conducted an intersection study to compare the YTHDF2-binding and interacted genes using a web tool (http://bioinformatics.psb.ugent.be/webtools/Venn/). Furthermore, we used the “clusterProfiler” R package to perform GO (Gene Ontology) enrichment analysis and KEGG (Kyoto encyclopedia of genes and genomes) pathway enrichment analysis on the combined two sets of results (Yu et al., 2012). Finally, the enriched pathways were visualized using the R packages “ggplot2” (https://cran.r-project.org/web/packages/ggplot2/index.html). In this case, R language software (R-3.6.3, 64-bit) (https://www.r-project.org/) was used.

Biologists and clinicians can use LinkedOmics to view, interpret, and compare cancer multi-omics data within and through tumor types (Vasaikar et al., 2018). The “LinkFinder” module was used to look for genes that had a strong relationship with YTHDF2. The “Link-Interpreter” module was used to investigate YTHDF2’s functions, pathways, and networks, as well as the functions, pathways, and networks of substantially associated genes. The simulation was set to 500 with a 0.05 *p*-value cutoff and a minimum number of genes (size) of 3. Gene set enrichment analysis was used to conduct the relevant tests (GO, KEGG pathways, kinase-target enrichment, transcription factor-target enrichment, and miRNA-target enrichment).

GeneMANIA is a website that allows us to create a protein-protein interaction (PPI) network and learn about the functions of submitted genes. GeneMANIA was used in this analysis to predict the role of genes that were enriched in KIRC (Warde-Farley et al., 2010).

CancerSEA is the first database devoted to decoding distinct functional states of cancer cells at the single-cell level (Yuan et al., 2019). In this case, we analyzed the functional status of YTHDF2 in the CancerSEA database to better understand the relevance and underlying mechanisms of YTHDF2 expression in KIRC.

Open Targets is a tool that uses a target-centric workflow to identify diseases that are possibly associated with a particular target, which was used in this analysis to detect diseases linked to YTHDF2 (Koscielny et al., 2017).

### Statistical Analyses

The Chi-square test was used to assess and evaluate the clinical and pathological conditions of the YTHDF2 low and high expression groups. The Cox logistic regression model was used to conduct univariate and multivariate analysis to find the independent prognostic variables that are significant for the prognosis of KIRC patients. Based on the result of multivariate cox regression analysis, we draw a nomogram by combining clinical factors and YTHDF2 gene expression to achieve a more reliable assessment of the 1-year, 3-years, and 5-years survival rate prediction of KIRC patients. R language software (R-3.6.3, 64-bit) (https://www.r-project.org/) was used to analyze the data and produce the nomogram. *p*-values less than 0.05 were deemed statistically significant.

## Results

### The Expression of YTHDF2 in KIRC

**FIGURE 1:**
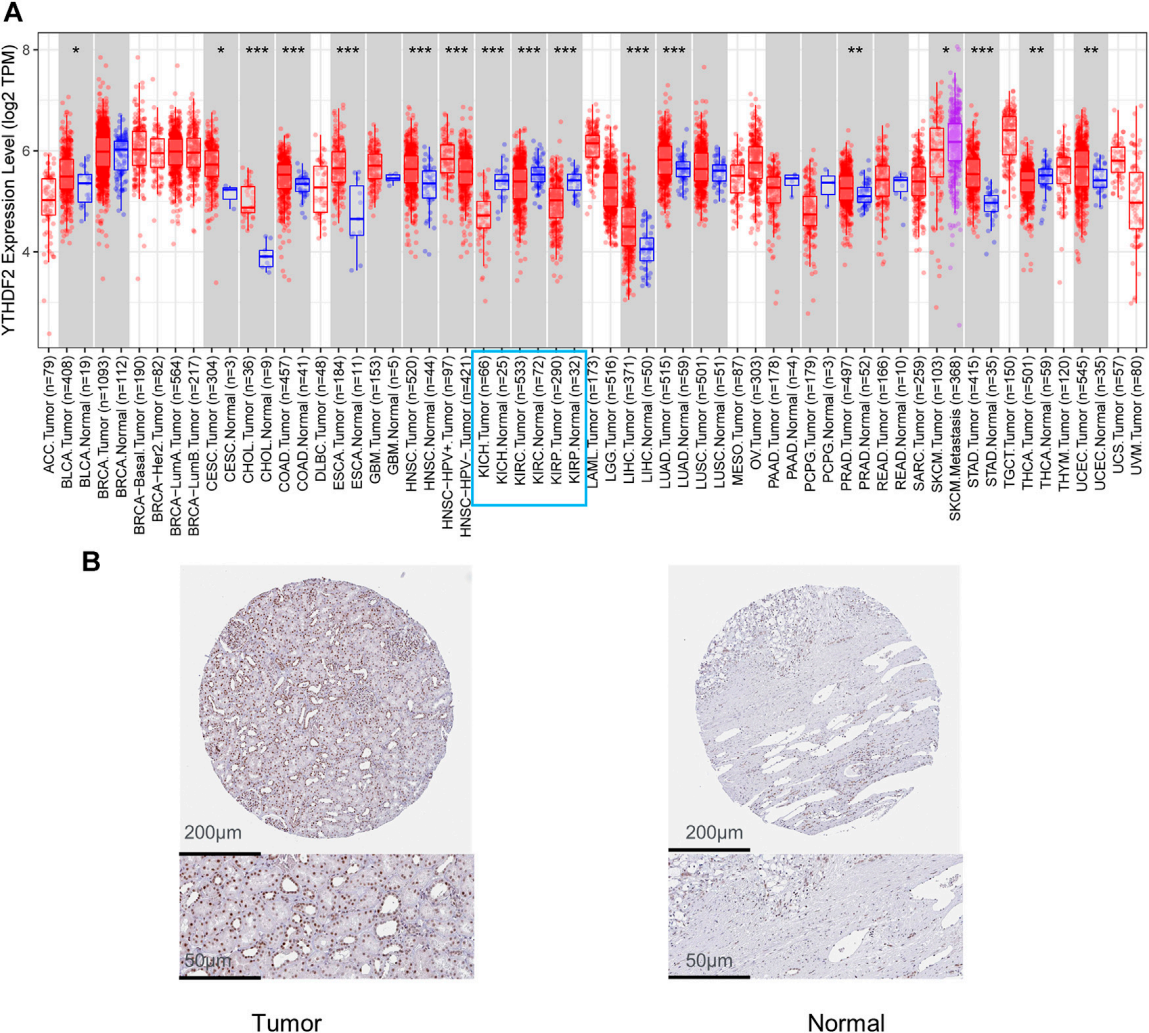
Expression level of YTHDF2 in renal clear cell carcinoma (KIRC). **(A)** YTHDF2 levels were shown to be higher or lower in various cancers in the TCGA database (TIMER). **(B)** Protein expression of YTHDF2 in KIRC. KIRC, Kidney renal clear cell carcinoma.

**FIGURE 2:**
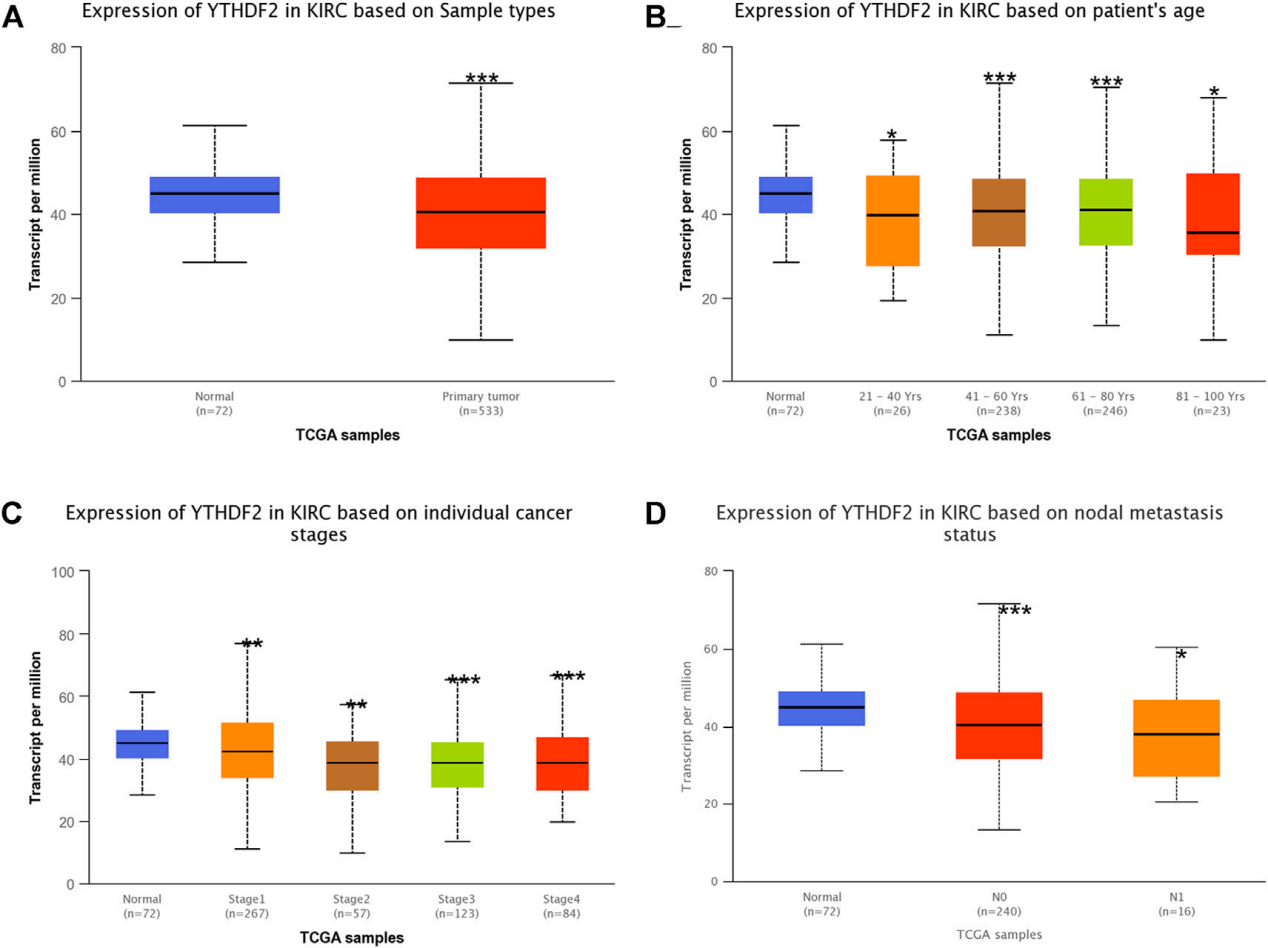
The expression of YTHDF2 mRNA in subgroups of KIRC patients (UALCAN). **(A)** The comparative expression of YTHDF2 in normal and KIRC samples. **(B)** YTHDF2 expression in KIRC patients aged 21–40, 41–60, 61–80, or 81–100. **(C)** Comparative expression of YTHDF2 in normal and KIRC (Stage 1, 2, 3, or 4) samples. **(D)** Expression of YTHDF2 in normal and KIRC (with or without nodal metastasis) samples. *, *p* < 0.05; **, *p* < 0.01; ***, *p* < 0.001. KIRC, Kidney renal clear cell carcinoma.

The TIMER2 method was used to examine the expression status of YTHDF2 across TCGA cancer forms (Figure 1A). In kidney renal clear cell carcinoma (KIRC), kidney chromophobe (KICH), kidney renal papillary cell carcinoma (KIRP), YTHDF2 expression in cancer tissues was lower than that in adjacent normal tissues. Furthermore, the primary role of YTHDF2 in KIRCs is unknown. As a result, we concentrated on YTHDF2’s position in the KIRC. We compared the expression level of the YTHDF2 protein in RCC patients to that in normal kidney tissues to determine the status of YTHDF2 expression. We found that YTHDF2 staining in RCC tissues was poorer than that in normal kidney tissues in the HPA (Figure 1B). In subgroup analyses based on sample gender, age, nodal metastasis status, and disease stage, the transcription level of YTHDF2 was significantly lower in RCC patients than in healthy individuals (Figure 2).

### Correlation Between Expression of YTHDF2 and Clinicopathological Parameters

Among the patients with renal clear carcinoma, 269 were in the YTHDF2 low expression group and 270 in the high YTHDF2 expression group. Correlation analysis revealed that YTHDF2 expression was significantly associated with T stage (*p* = 0.011) and histologic grade (*p* = 0.002). There were no significant differences in pathologic stage, age, indicators of metastasis to regional lymph nodes, presence of distant metastasis, serum calcium, and hemoglobin levels between the two groups (Supplementary Table S1).

**FIGURE 3:**
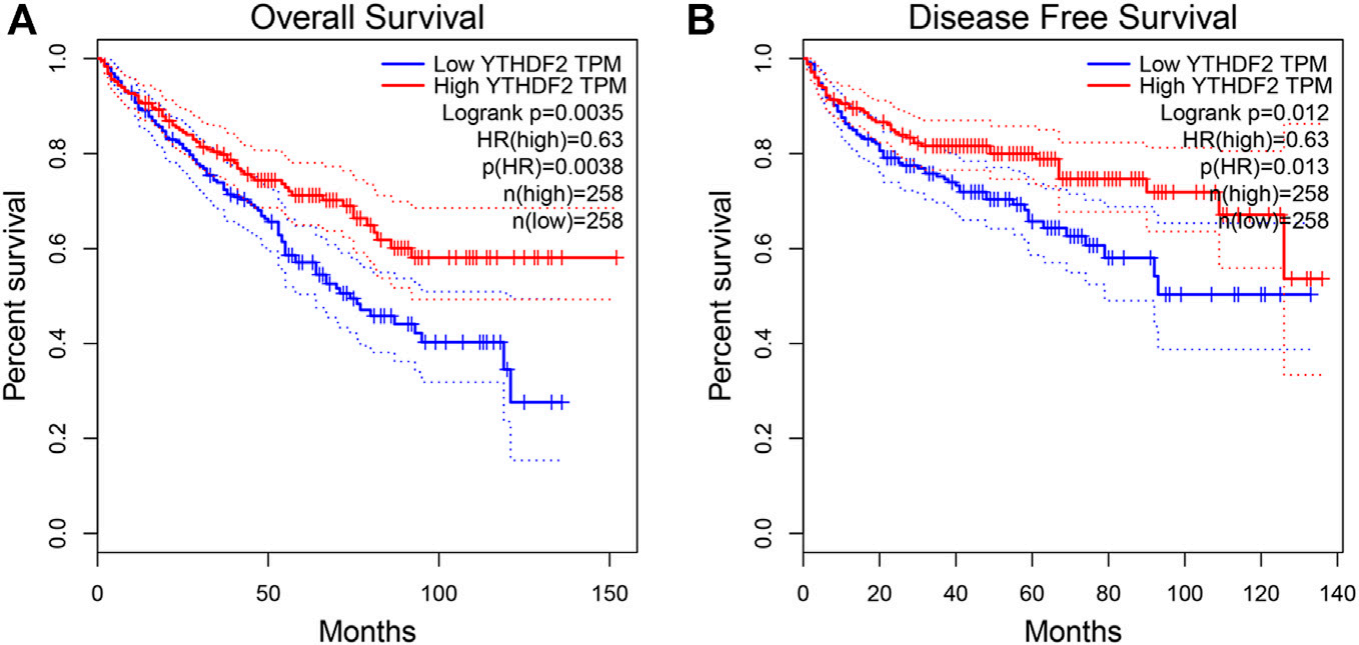
The prognostic value of YTHDF2 in KIRC. **(A)** The disease-free survival in KIRC patients with high or low YTHDF2 expression (GEPIA2). **(B)** The overall survival in KIRC patients with high or low expression of YTHDF2 (GEPIA2). KIRC, Kidney renal clear cell carcinoma. KIRC, Kidney renal clear cell carcinoma.

To ascertain the prognostic value of YTHDF2 in KIRC, GEPIA2 was utilized to analyze the relationship between YTHDF2 expression and clinical follow-up data, and the log-rank statistical method was used for significance tests. The results showed that the elevated expression of YTHDF2 was positively correlated with disease-free survival (DFS) and overall survival (OS), indicating that the KIRC patients with low expression of YTHDF2 were correlated with a poor prognosis (*n* = 516, *p* < 0.05, Figure 3).

**FIGURE 4:**
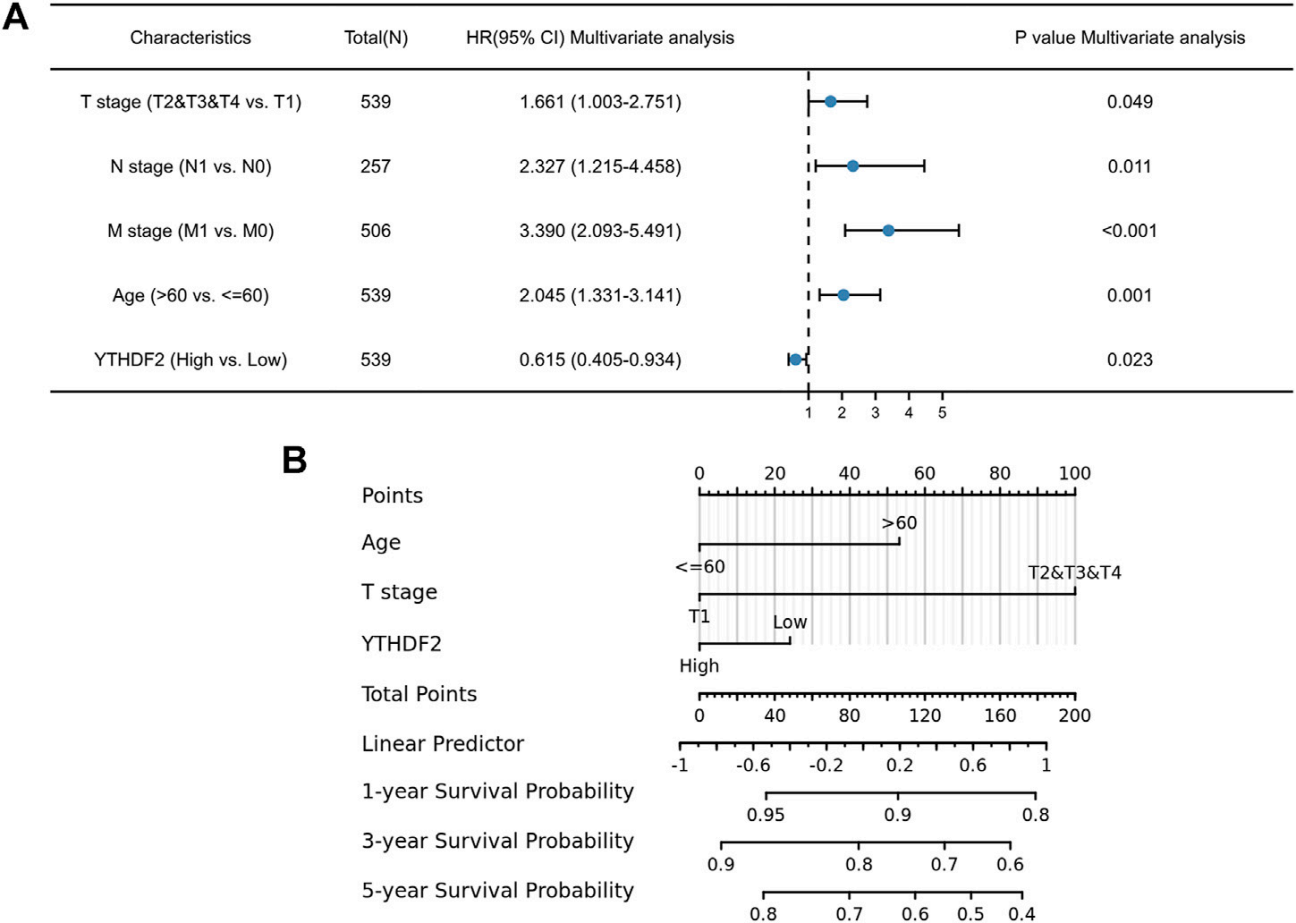
Forest plot and nomogram construction. **(A)** Forest plot of the association between risk factors and overall survival (OS) in TCGA-KIRC patients. **(B)** Prediction model of nomogram construction. KIRC, Kidney renal clear cell carcinoma.

To further identify the risk factors associated with OS in patients with RCC, univariate, and multivariate analyses were performed to determine whether low expression of YTHDF2 is an independent risk factor for poor prognosis. Univariate analysis showed that prognosis was related to the age, clinical stage, T stage, N stage, M stage, pathologic stage, and YTHDF2 expression (Supplementary Table S2). Multivariate analysis showed that YTHDF2 expression was an independent risk factor for tumor progression (TCGA-KIRC cohort: OS, HR = 0.615, 95% CI: 0.405–0.934; *p* < 0.05) (Figure 4A).

These data suggest that elevated YTHDF2 expression significantly prolongs OS and PFS in patients with KIRC. To obtain a more accurate prediction of survival rate in KIRC patients, we constructed a nomogram by integrating clinical factors and gene expression of YTHDF2 (Figure 4B).

### Correlationship Between Expression of YTHDF2 and KIRC Immune Biomarkers

**FIGURE 5:**
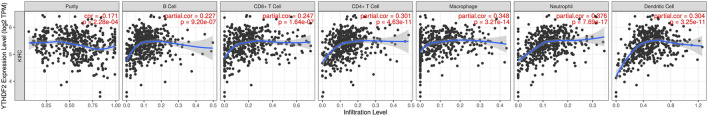
The expression of YTHDF2 was shown to be significantly linked to immune cell infiltration.

Compelling data have shown a strong association between tumor-infiltrating lymphocytes and cancer survival (Zhu et al., 2017; Denkert et al., 2018; Corredor et al., 2019). Consequently, we examined whether the expression of YTHDF2 was associated with TIMER levels during immune infiltration in KIRC. B cells (Cor = 0.227, *p* = 2.03e-03), CD8^+^ T cells (Cor = 0.247, *p* = 1.64e-07), CD4^+^ T cells (Cor = 0.301, *p* = 4.63e-11), macrophages (Cor = 0.348, *p* = 3.21e-14), neutrophils (Cor = 0.376, *p* = 7.69e-17), and dendritic cells (Cor = 0.304, *p* = 3.25e-11) were positively associated with YTHDF2 expression (Figure 5). The relationship between differentially expressed YTHDF2 and immune cell infiltration was also investigated. The Cox proportional hazard model was used, and confounding variables such as B cells, CD4^+^ T cells, macrophages, neutrophils, and YTHDF2 were considered. CD8^+^ T cells (*p* = 0.014), macrophages (*p* = 0.012), neutrophil cells (*p* = 0.016), and YTHDF2 expression (*p* = 0) were both shown to be consistent with the treatment outcomes in RCC patients (Supplementary Table S3).

### Genetic Alternation, Functional States in the scRNA-Seq Datasets, Enrichment Analysis of YTHDF2-Related Partners, and Genetic Alteration in KIRC

Using cBioPortal, we ascertained the genetic modification status of YTHDF2 in TCGA-KIRC. We discovered that YTHDF2 was altered in 32 of 537 (6%) patients with KIRC, with mRNA upregulation in 17 cases (3.2%) and mutation in 15 cases (2.8%). (Supplementary Figure S1A). We were unable to find a connection between YTHDF2 mutations and KIRC OS and disease-free survival (DFS) prognosis (Supplementary Figures S1B,C, both *p* > 0.05).

**FIGURE 6:**
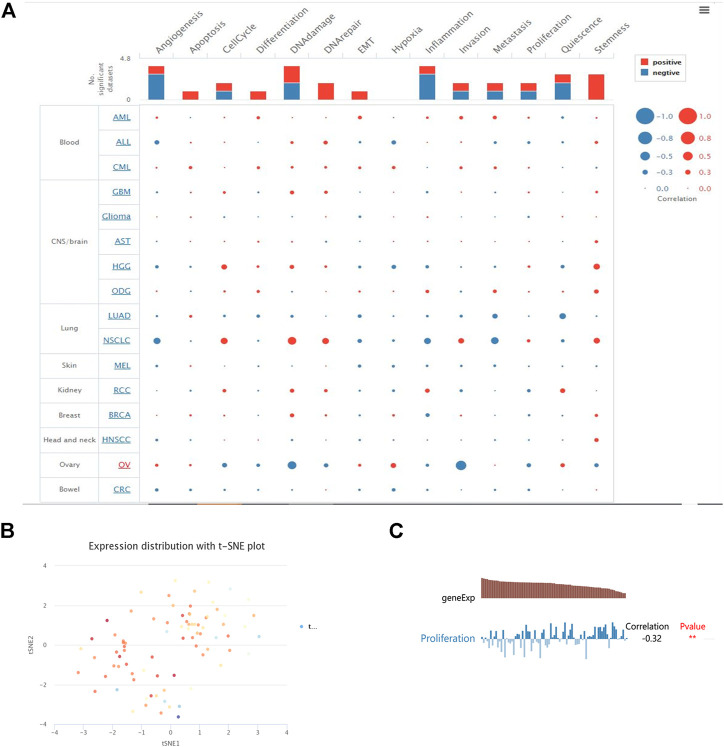
YTHDF2 functional states in the scRNA-seq datasets. **(A)** Relevance of YTHDF2 across 14 functional states in distinct cancers. **(B)** The overall expression of the module in kidney clear carcinoma was found to be heterogeneous. **(C)** Correlations between the YTHDF2 expression and two functional states were identified by the CancerSEA database. * *p* < 0.05, **, *p* < 0.01, *** *p* < 0.001.

We examined the functional status of YTHDF2 across various cancer types in the CancerSEA database to better understand the relevance and underlying mechanisms of YTHDF2 expression in cancer. An interactive bubble map was used to investigate the expression of YTHDF2 and the behavior of each functional state using single-cell datasets for various cancers. An overview of the relationship between the functional state and the number of single-cell datasets is shown in the upper bar plot (Figure 6A). According to the clustering results of CancerSEA, the overall expression of the module in the KIRC was found to be heterogeneous (Figure 6B). YTHDF2 was negatively correlated with proliferation in KIRC (Figure 6C).

**FIGURE 7:**
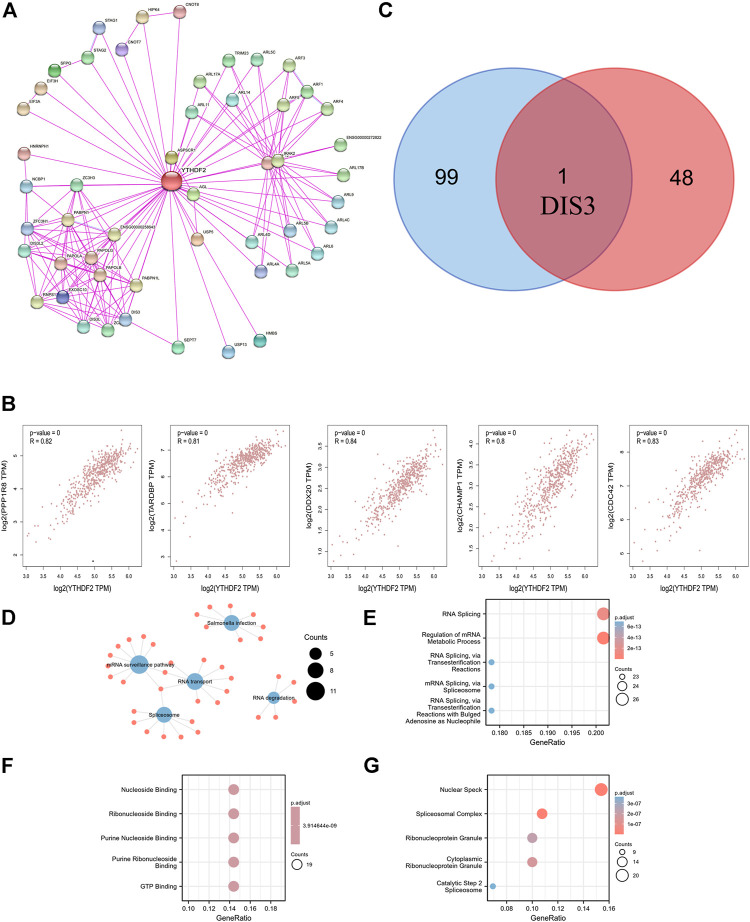
YTHDF2-related gene enrichment analysis and GO and KEGG analysis. **(A)** The available experimentally determined YTHDF2-binding proteins using the STRING tool. **(B)** The expression correlation between YTHDF2 and selected targeting genes, including PPP1R8, TARDBP, DDX20, CHAMP1 and CDC42. **(C)** An intersection analysis of the-binding and correlated genes was conducted. **(D)** KEGG pathway analysis. **(E)** Biological processes. **(F)** Molecular functions. **(G)** Cellular components.

**FIGURE 8:**
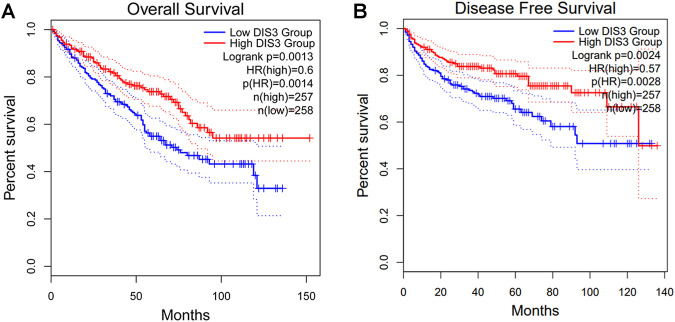
The survival analysis of DIS3 in KIRC. KIRC, Kidney renal clear cell carcinoma.

We attempted to screen out the targeting YTHDF2-binding proteins and the YTHDF2 expression-correlated genes for a series of pathway enrichment studies to learn more about the molecular function of the YTHDF2 gene in tumorigenesis. We identified 50 YTHDF2-binding proteins using the STRING instrument, all of which were confirmed by experimental data (Supplementary Table S4). The interaction network of these proteins was investigated (Figure 7A). We combined TCGA KIRC expression data with the GEPIA2 method to identify the top 100 genes associated with YTHDF2 expression (Supplementary Table S5). The expression of YTHDF2 was found to be positively associated with that of DDX20 (DEAD-box helicase 20) (R = 0.84), CDC42 (Cell Division Cycle 42) (R = 0.83), TARDBP (TAR DNA binding protein) (R = 0.81), PPP1R8 (Protein Phosphatase 1 Regulatory Subunit 8) (R = 0.82), and CHAMP1 (Chromosome Alignment Maintaining Phosphoprotein 1) (R = 0.8) genes (all *p* < 0.001) (Figure 7B). An intersection analysis of the two groups revealed that they shared one similar member, DIS3 (DIS3 homolog, exosome endoribonuclease, and 3′–5′ exoribonuclease) (Figure 7C). High DIS3 expression was linked to a positive prognosis in KIRC, according to GEPIA2 (Figure 8).

To do KEGG and GO enrichment tests, we merged those two datasets. The KEGG data suggest that the “mRNA surveillance pathway,” “RNA degradation,” and “RNA transport” might be involved in the effect of YTHDF2 on tumorigenesis and progression of KIRC (Figure 7D). Most of these genes are related to pathways or cellular biology of RNA metabolism and nucleoside binding, such as regulation of mRNA metabolic process, RNA splicing, ribonucleoside binding, purine ribonucleoside binding, nuclear speck, and spliceosomal complex, according to the GO enrichment analysis results (Figures 7E–G).

We submitted YTHDF2 to Open Targets, a forum that provides a target-centric workflow for identifying diseases that may be associated with a given target to further validate its role. YTHDF2 is involved in immune and urinary system diseases (Supplementary Figure S2). These data suggest that YTHDF2 may play a role in immune escape in the KIRC microenvironment.

### YTHDF2 Kinase, miRNA, and Transcription Factor Target Networks in KIRC

Since YTHDF2 is essential in KIRC, we utilized LinkedOmics and GSEA to identify targets of THDF2 in KIRC. YTHDF2 kinase, miRNA, and transcription factor target networks in the KIRC were investigated. The kinases BCR, ROCK, MKNK1, PRKC, and MKNK2 were the top five most significant kinase targets (Supplementary Figure S3A). The PPI network generated using GeneMANIA and correlated kinase BCR genes showed that these gene sets were primarily responsible for protein insertion into the membrane in the apoptotic signaling pathway (Supplementary Figure S4). MIR-202 (ATAGGAA) and MIR-381 (CTTGTAT) were significant miRNA targets (Supplementary Figure S3B). The gene sets responsible for negative regulation of growth, regulation of cell junction assembly, regulation of growth, negative regulation of cell growth, and post-transcriptional regulation of gene expression were identified in the PPI network generated using GeneMANIA with correlated genes of MIR-202 (ATAGGAA) (Supplementary Figure S5). V$E2F Q2, V$ETF Q6, V$MYC Q2, V$USF2 Q6, and V$TAXCREB 02 were often associated with YTHDF2’s transcription factor-target network (Supplementary Figure S3C). Furthermore, the PPI network generated using GeneMANIA with V$E2F Q2 correlated genes showed that these gene sets were primarily responsible for the PcG protein complex, PRC1 complex, histone monoubiquitination, histone ubiquitination, protein monoubiquitination, and nuclear ubiquitin ligase complex (Supplementary Figure S6).

## Discussion

YTHDF2, a member of the binding protein of m6A modification, the most widely distributed RNA modification in eukaryotes (Meyer and Jaffrey, 2017; Ji et al., 2018).

YTHDF2, one of the m6A “readers,” binds to m6A-modified mRNA and destabilizes it (Du et al., 2016; Ivanova et al., 2017). Accumulating evidence has shown that YTHDF2 is engaged in the development and progression of tumors (Chen et al., 2017, 2018, 2; Paris et al., 2019). The role of YTHDF2 in the pathogenesis of KIRC has not yet been characterized. Therefore, we used bioinformatics analysis of public sequencing data to direct future research in KIRC to obtain more comprehensive insights into the possible roles of YTHDF2 in KIRC.

Results from our study revealed downregulated mRNA and protein expression of YTHDF2 in KIRC. Subgroup analysis based on sample type, age, lymph node metastasis status, and disease stage showed that the level of YTHDF2 transcription in KIRC patients was substantially lower than that in healthy individuals. Correlation analysis showed that YTHDF2 expression was significantly associated with T stage (*p* = 0.011) and histologic grade (*p* = 0.002). Interestingly, we used the GEPIA2 tool to detect that patients with high YTHDF2 expression showed higher OS and DFS than those with low YTHDF2 expression.

A multivariate Cox study indicated that YTHDF2 expression was a predictor of OS in patients with KIRC. The clinicopathologic features associated with poor KIRC prognosis include age, N stage, M stage, and YTHDF2 expression, as suggested by the forest plot (Figure 4A). A predictive nomogram for KIRC that combines YTHDF2 expression with the above clinical variables has not yet been formulated. Based on the results of Cox regression analysis, we constructed a nomogram by integrating clinical factors and gene expression of YTHDF2 to obtain a more accurate assessment of the 1-year, 3-years, and 5-years survival rate prediction of KIRC patients. This also has practical significance for determining patient prognosis. In the internal validation, the c-index of the nomogram was 0.758 (95% CI, 0.854–0.941), which indicates that the prediction model has relatively good accuracy.

Cancer immunotherapy has recently been demonstrated to be effective and important for tumor treatment (Farkona et al., 2016). In pan-cancer distinctions, renal cell carcinoma is one of the most immune-infiltrated tumors (Şenbabaoğlu et al., 2016). In reality, it is critical to understand the immune infiltration state of cancer patients to choose the best immunotherapy strategy for each condition. We used TIMER to explore whether YTHDF2 expression is associated with KIRC immune infiltration. Our findings show that YTHDF2 expression is associated with various immune marker sets and stages of immune infiltration in KIRC. Our findings demonstrate that YTHDF2 expression is significantly correlated with the infiltration level of B cells, CD8^+^ T cells (Cor = 0.247, *p* = 1.64e-07), CD4^+^ T cells (Cor = 0.301, *p* = 4.63e-11), macrophages (Cor = 0.348, *p* = 3.21e-14), neutrophils (Cor = 0.376, *p* = 7.69e-17), and dendritic cells. According to Open Targets evidence, YTHDF2 plays an important role in the immune system and urinary system diseases. As a result, we hypothesized that YTHDF2 could influence tumor immunology and serve as an immunotherapeutic target for KIRC therapy. According to the scRNA data of CancerSEA, YTHDF2 was negatively correlated with proliferation in RCC. These results were consistent with the previous survival analysis.

We combined data on YTHDF2-binding components and expression-related genes for a series of enrichment studies and discovered the possible role of “mRNA surveillance pathway,” “RNA degradation,” and “RNA transport” in the etiology or pathogenesis of KIRC. The intersection analysis of STRING and GEPIA2 showed one common member, DIS3. The protein product of DIS3 is a part of the exosome complex in the nucleus of eukaryotic cells (Tomecki et al., 2010). DIS3 performs a variety of RNA metabolism roles, including mRNA quality control, gene expression regulation, and minor RNA processing (Robinson et al., 2015, 3). Failure to maintain a balance between the synthesis and degradation of RNA in the cell may lead to major changes in cell function. From the perspective of cancer progression, it can be speculated that DIS3 will enable cancer cells to regulate the expression of specific genes through means such as RNA degradation and other means. As a result, we examined DIS3 expression in KIRC tissues and non-tumor tissues and observed that DIS3 expression in KIRC tissues was slightly lower than in non-tumor tissues. High expression of DIS3 was correlated with a positive prognosis in KIRC, according to the survival data. We hypothesize that YTHF2 and DIS3 could collaborate in the production and advancement of KIRC.

The most significant kinases, miRNA, and transcription factor-target network of YTHDF2 in KIRC are associated with kinase BCR, MIR-202 (ATAGGAA), and V$E2F_Q2. The PPI network built with GeneMANIA and correlated genes of kinases BCR and MIR-202 (ATAGGAA) indicated that these gene sets were primarily responsible for apoptotic signaling pathways and negative growth regulation. Via BCR kinase and MIR-202, YTHDF2 can participate in the apoptotic signaling pathway and negatively regulate growth, influencing the occurrence and production of KIRC.

E2F transcription factors regulate the expression of several genes involved in cell proliferation, especially those involved in cell cycle progression through the G1 and S phases (Nevins, 2001). The Rb/E2F pathway regulates cell cycle initiation, proliferation, and apoptosis (Nevins, 2001). Disrupting the control of this pathway occurs in virtually all cancers and leads to increased activity of oncogenic E2F, leading to uncontrolled proliferation (Kent and Leone, 2019). TCGA-KIRC was used to analyze the expression levels of the E2F family genes in normal tissues and tumor tissues, and the results revealed that the majority of the E2F family genes were upregulated. (Wang H. et al., 2020).

Thus, our findings indicate that E2F is a key target of YTHDF2 and that YTHDF2 regulates the cell cycle and proliferation potential through this factor. Given the paucity of appropriate research, further research should be conducted to confirm this theory.

There were some limitations to our study. First, all data analyzed in this study were retrieved from online databases, further experiments and clinical trials are needed to confirm the value of YTHDF2 in KIRC Second, because the appropriate m6A sequencing data was incomplete, we did not merge transcriptome sequencing and m6A sequencing data for bioinformatics research. We did not assess the potential diagnostic roles of YTHDF2 in KIRC; therefore, future studies are needed to explore whether YTHDF2 could be used as a diagnostic marker. Experiments are needed to better understand the role and mechanism of the YTHDF2/E2F axis in KIRC.

## Conclusion

In conclusion, our study shows that YTHDF2 is significantly downregulated and is associated with immune infiltration in KIRC. High YTHDF2 expression was linked to a positive prognosis and could be used as an independent prognostic predictor in patients with KIRC. In addition, YTHDF2 may regulate cell proliferation through the apoptosis pathway *via* E2F. Overall, further research is needed to elucidate the precise molecular mechanism that will aid in the advancement of KIRC-targeted therapy.

## Data Availability

The original contributions presented in the study are included in the article/Supplementary Material, further inquiries can be directed to the corresponding authors.
