# Supplementary figures and images for: YTHDF2 is a Potential Biomarker and Associated with Immune Infiltration in Kidney Renal Clear Cell Carcinoma
Source: Front Pharmacol. 2021 Aug 27;12:709548. doi: 10.3389/fphar.2021.709548 (PMC8429956; doi:10.3389/fphar.2021.709548)

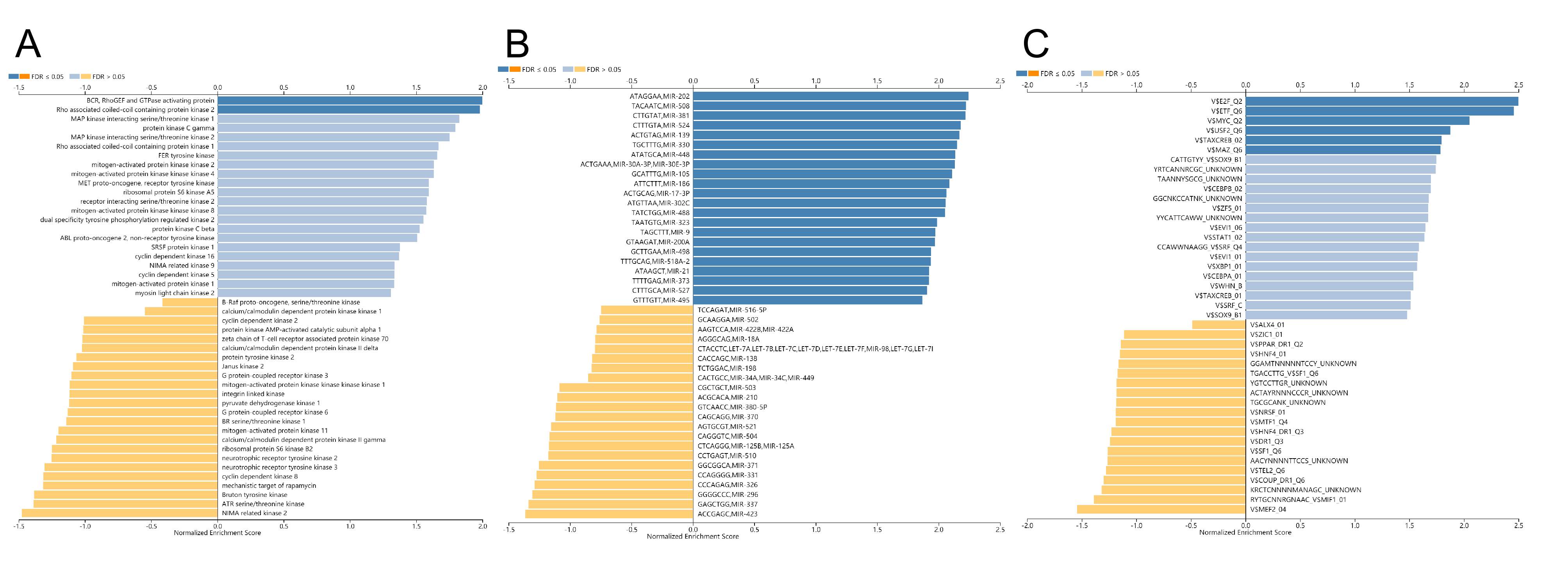

Supplement: Supplementary file 1 [file Image3.TIFF]

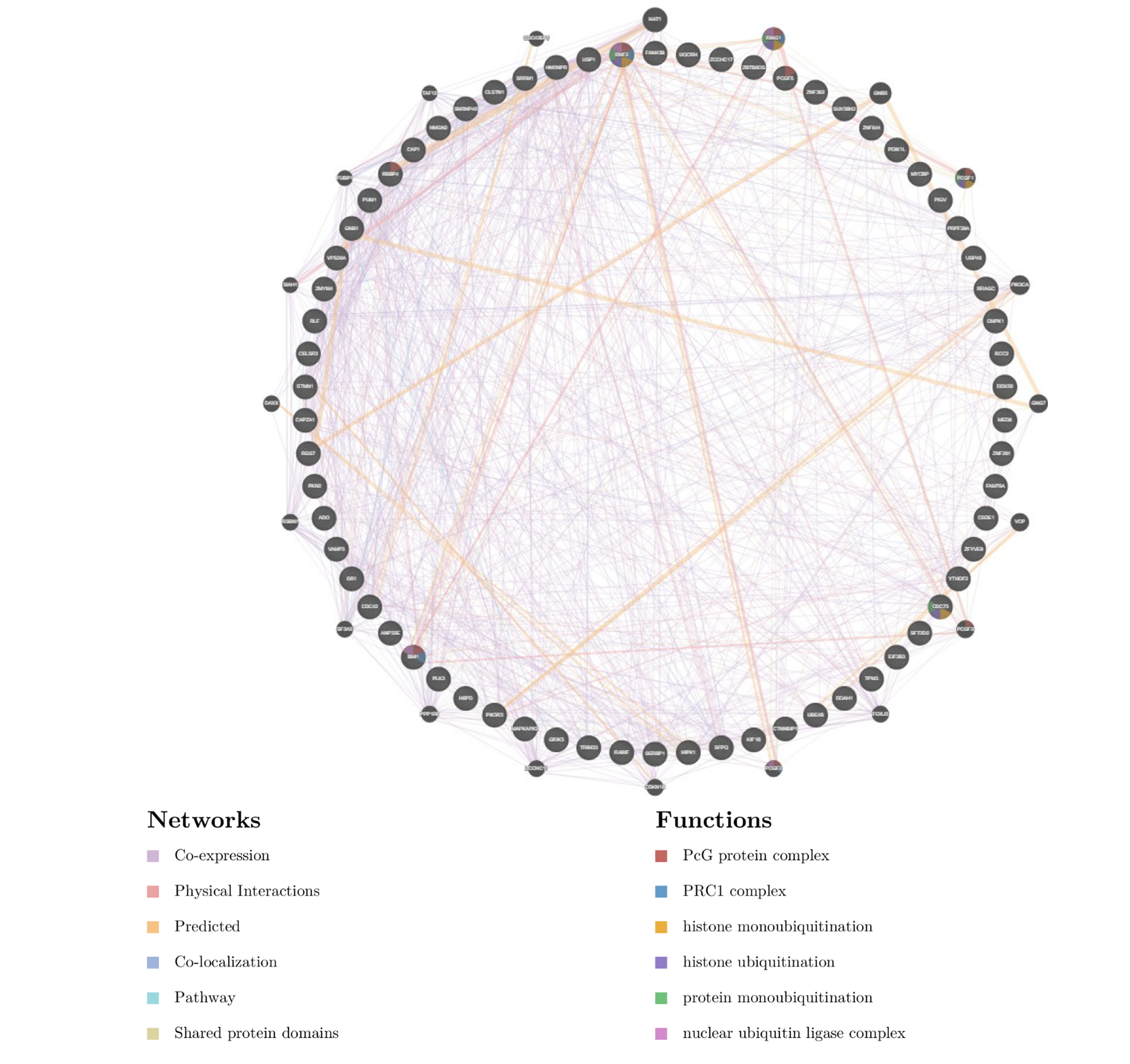

Supplement: Supplementary file 2 [file Image6.TIF]

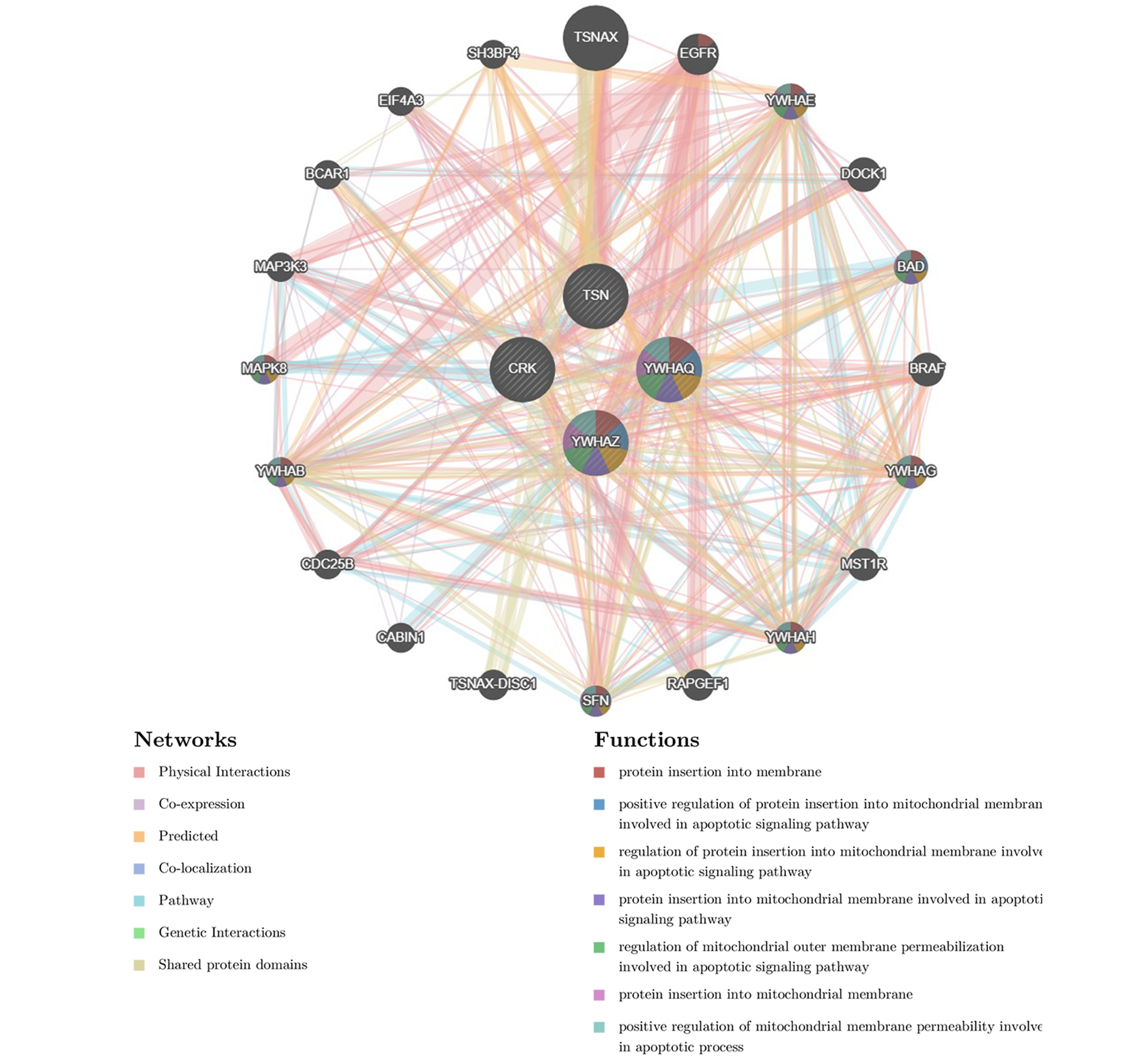

Supplement: Supplementary file 4 [file Image4.TIF]

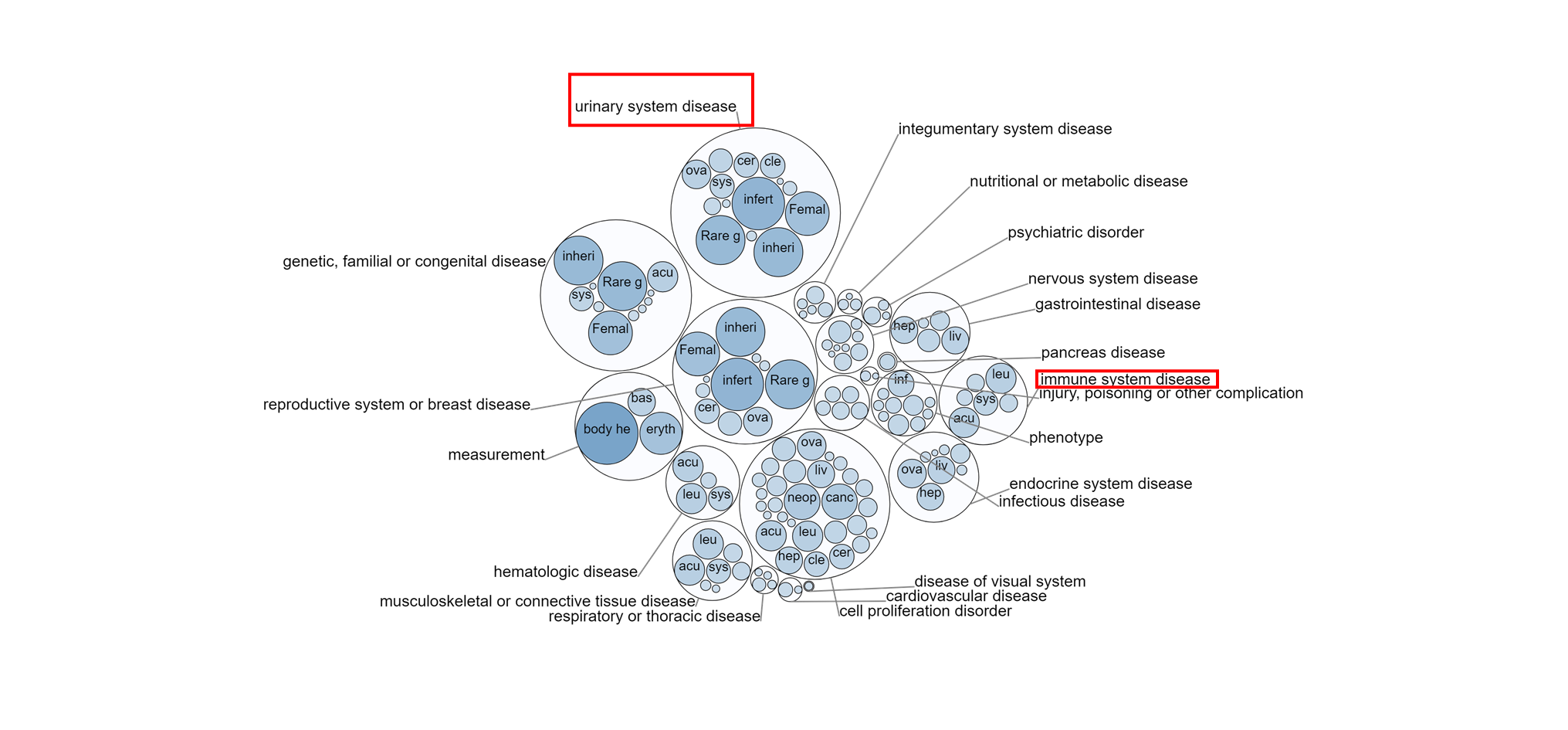

Supplement: Supplementary file 5 [file Image2.TIF]

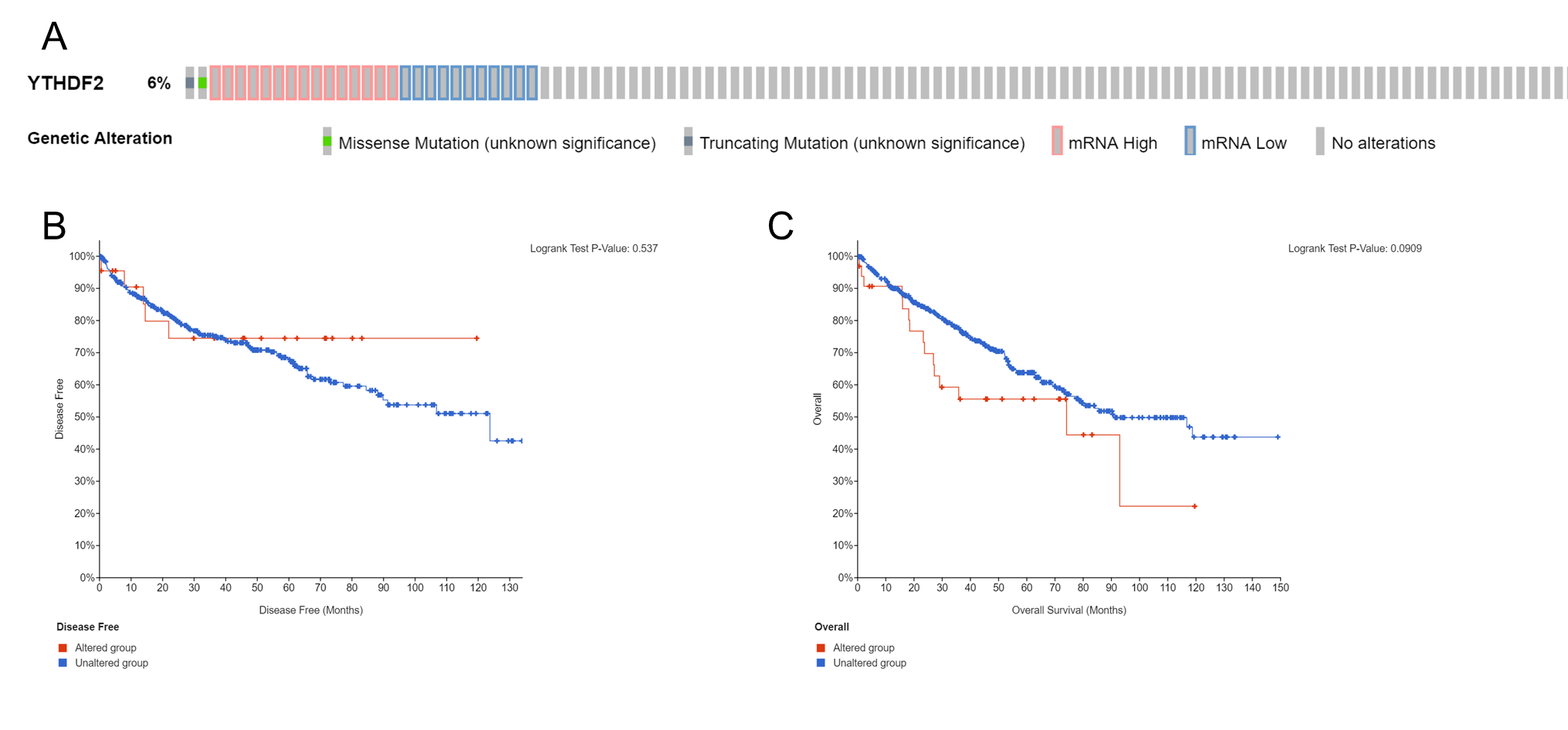

Supplement: Supplementary file 6 [file Image1.TIF]

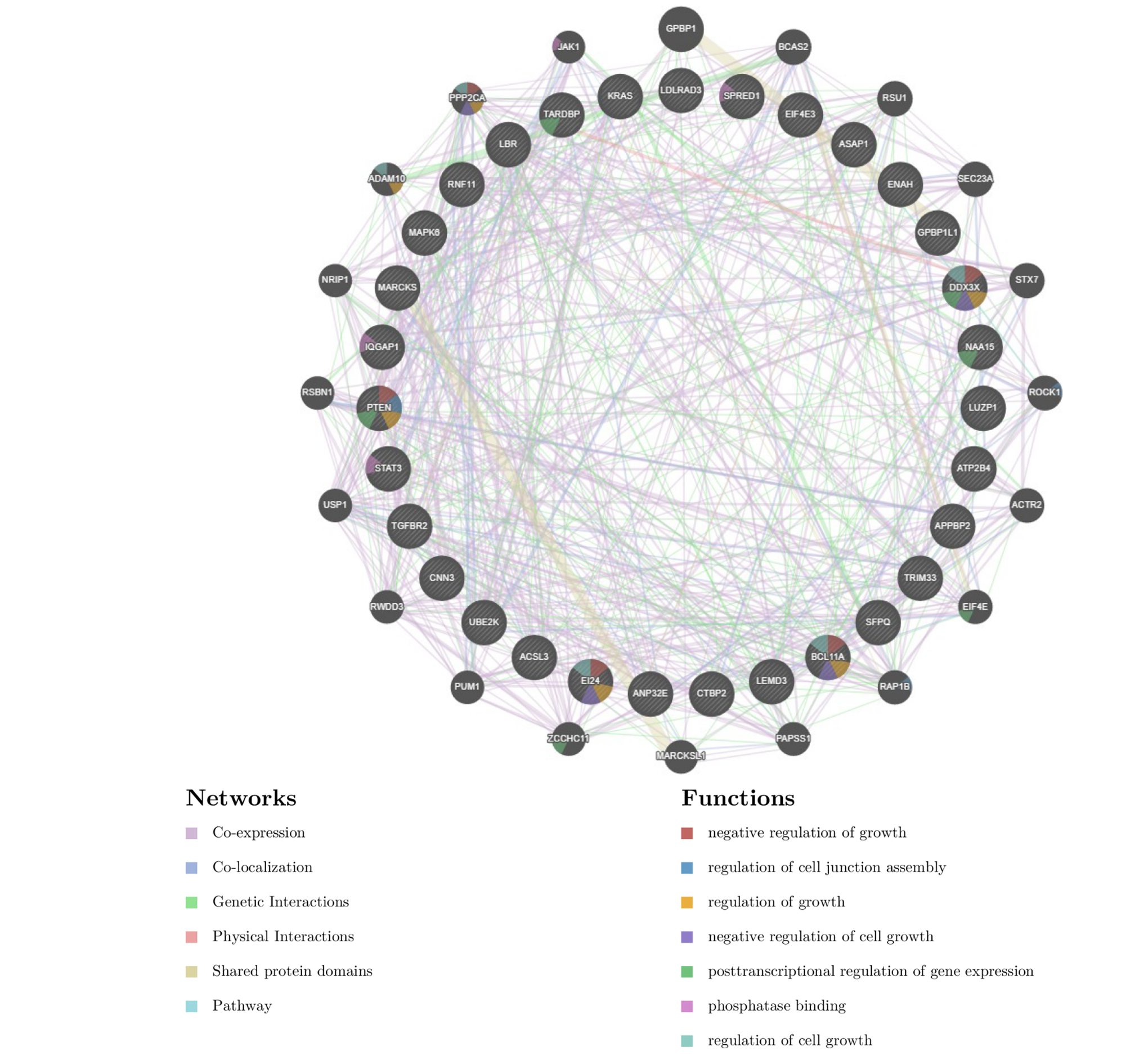

Supplement: Supplementary file 10 [file Image5.TIF]
