# Supplementary material for: YTHDF2 is a Potential Biomarker and Associated with Immune Infiltration in Kidney Renal Clear Cell Carcinoma
Source: Front Pharmacol. 2021 Aug 27;12:709548. doi: 10.3389/fphar.2021.709548 (PMC8429956; doi:10.3389/fphar.2021.709548)
Supplement: Supplementary file 3 [file Table1.DOCX]

Supplementary Table 1 The Relationship Between the Expression of YTHDF2 and Clinicopathological Data

| Characteristic | levels | Low expression of YTHDF2 | High expression of YTHDF2 | p |
| --- | --- | --- | --- | --- |
| n |  | 269 | 270 |  |
| T stage, n (%) | T1 | 124 (23%) | 154 (28.6%) | 0.011 |
|  | T2 | 44 (8.2%) | 27 (5%) |  |
|  | T3 | 98 (18.2%) | 81 (15%) |  |
|  | T4 | 3 (0.6%) | 8 (1.5%) |  |
| N stage, n (%) | N0 | 118 (45.9%) | 123 (47.9%) | 1.000 |
|  | N1 | 8 (3.1%) | 8 (3.1%) |  |
| M stage, n (%) | M0 | 209 (41.3%) | 219 (43.3%) | 0.268 |
|  | M1 | 44 (8.7%) | 34 (6.7%) |  |
| Pathologic stage, n (%) | Stage I | 121 (22.6%) | 151 (28.2%) | 0.092 |
|  | Stage II | 34 (6.3%) | 25 (4.7%) |  |
|  | Stage III | 67 (12.5%) | 56 (10.4%) |  |
|  | Stage IV | 45 (8.4%) | 37 (6.9%) |  |
| Age, n (%) | <=60 | 135 (25%) | 134 (24.9%) | 0.966 |
|  | >60 | 134 (24.9%) | 136 (25.2%) |  |
| Histologic grade, n (%) | G1 | 6 (1.1%) | 8 (1.5%) | 0.002 |
|  | G2 | 96 (18.1%) | 139 (26.2%) |  |
|  | G3 | 119 (22.4%) | 88 (16.6%) |  |
|  | G4 | 43 (8.1%) | 32 (6%) |  |
| Serum calcium, n (%) | Elevated | 6 (1.6%) | 4 (1.1%) | 0.880 |
|  | Low | 102 (27.9%) | 101 (27.6%) |  |
|  | Normal | 78 (21.3%) | 75 (20.5%) |  |
| Hemoglobin, n (%) | Elevated | 3 (0.7%) | 2 (0.4%) | 0.248 |
|  | Low | 142 (30.9%) | 121 (26.4%) |  |
|  | Normal | 89 (19.4%) | 102 (22.2%) |  |
|  |  |  |  |  |
