# Supplementary material for: YTHDF2 is a Potential Biomarker and Associated with Immune Infiltration in Kidney Renal Clear Cell Carcinoma
Source: Front Pharmacol. 2021 Aug 27;12:709548. doi: 10.3389/fphar.2021.709548 (PMC8429956; doi:10.3389/fphar.2021.709548)
Supplement: Supplementary file 7 [file Table2.DOCX]

Supplementary Table 2 Cox regression analysis the association between clinicopathological variables and overall survival.

| **Characteristics** | **Total(N)** | **Univariate analysis** | |
| --- | --- | --- | --- |
|  |  | Hazard ratio (95% CI) | P value |
| T stage (T2&T3&T4 vs. T1) | 539 | 2.917 (2.095-4.061) | <0.001 |
| N stage (N1 vs. N0) | 257 | 3.453 (1.832-6.508) | <0.001 |
| M stage (M1 vs. M0) | 506 | 4.389 (3.212-5.999) | <0.001 |
| YTHDF2 (High vs. Low) | 539 | 0.718 (0.532-0.971) | 0.031 |
| Pathologic stage (Stage II&Stage III&Stage IV vs. Stage I) | 536 | 3.299 (2.342-4.648) | <0.001 |
| Gender (Male vs. Female) | 539 | 0.930 (0.682-1.268) | 0.648 |
| Age (>60 vs. <=60) | 539 | 1.765 (1.298-2.398) | <0.001 |
| Histologic grade (G2&G3&G4 vs. G1) | 531 | 9231340.001 (0.000-Inf) | 0.992 |
