# Supplementary material for: YTHDF2 is a Potential Biomarker and Associated with Immune Infiltration in Kidney Renal Clear Cell Carcinoma
Source: Front Pharmacol. 2021 Aug 27;12:709548. doi: 10.3389/fphar.2021.709548 (PMC8429956; doi:10.3389/fphar.2021.709548)
Supplement: Supplementary file 9 [file Table3.DOCX]

Supplementary Table 3 The Cox proportional hazard model of YTHDF2 and six tumor-infiltrating immune cells in RCC (TIMER).

|  | **coef** | **HR** | **95%CI_l** | **95%CI_u** | **p-value** | **sig** |
| --- | --- | --- | --- | --- | --- | --- |
| **B_cell** | -0.777 | 0.460 | 0.022 | 9.472 | 0.615 |  |
| **CD8_Tcell** | -1.918 | 0.147 | 0.032 | 0.676 | 0.014 | * |
| **CD4_Tcell** | -0.525 | 0.592 | 0.046 | 7.545 | 0.686 |  |
| **Macrophage** | -2.898 | 0.055 | 0.006 | 0.532 | 0.012 | * |
| **Neutrophil** | 4.718 | 111.92 | 2.374 | 5276.843 | 0.016 | * |
| **Dendritic** | 1.641 | 5.161 | 0.959 | 27.757 | 0.056 | · |
| **YTHDF2** | -0.722 | 0.486 | 0.335 | 0.705 | 0 | *** |
